# Microalgae biofuels: illuminating the path to a sustainable future amidst challenges and opportunities

**DOI:** 10.1186/s13068-024-02461-0

**Published:** 2024-01-23

**Authors:** Min Wang, Xiaoxue Ye, Hongwen Bi, Zhongbao Shen

**Affiliations:** 1 Institute of Agricultural Remote Sensing and Information, Heilongjiang Academy of Agricultural Sciences, Harbin, 150086 China; 2 Sanya Research Institute of Chinese Academy of Tropical Agricultural Sciences, Sanya, 572025 China; 3 Grass and Science Institute of Heilongjiang Academy of Agricultural Sciences, Harbin, 150086 China

**Keywords:** Microalgae biofuels, Lipid, Selection strategies, PBRs, Genetic engineering

## Abstract

The development of microalgal biofuels is of significant importance in advancing the energy transition, alleviating food pressure, preserving the natural environment, and addressing climate change. Numerous countries and regions across the globe have conducted extensive research and strategic planning on microalgal bioenergy, investing significant funds and manpower into this field. However, the microalgae biofuel industry has faced a downturn due to the constraints of high costs. In the past decade, with the development of new strains, technologies, and equipment, the feasibility of large-scale production of microalgae biofuel should be re-evaluated. Here, we have gathered research results from the past decade regarding microalgae biofuel production, providing insights into the opportunities and challenges faced by this industry from the perspectives of microalgae selection, modification, and cultivation. In this review, we suggest that highly adaptable microalgae are the preferred choice for large-scale biofuel production, especially strains that can utilize high concentrations of inorganic carbon sources and possess stress resistance. The use of omics technologies and genetic editing has greatly enhanced lipid accumulation in microalgae. However, the associated risks have constrained the feasibility of large-scale outdoor cultivation. Therefore, the relatively controllable cultivation method of photobioreactors (PBRs) has made it the mainstream approach for microalgae biofuel production. Moreover, adjusting the performance and parameters of PBRs can also enhance lipid accumulation in microalgae. In the future, given the relentless escalation in demand for sustainable energy sources, microalgae biofuels should be deemed a pivotal constituent of national energy planning, particularly in the case of China. The advancement of synthetic biology helps reduce the risks associated with genetically modified (GM) microalgae and enhances the economic viability of their biofuel production.

**Graphical Abstract:**

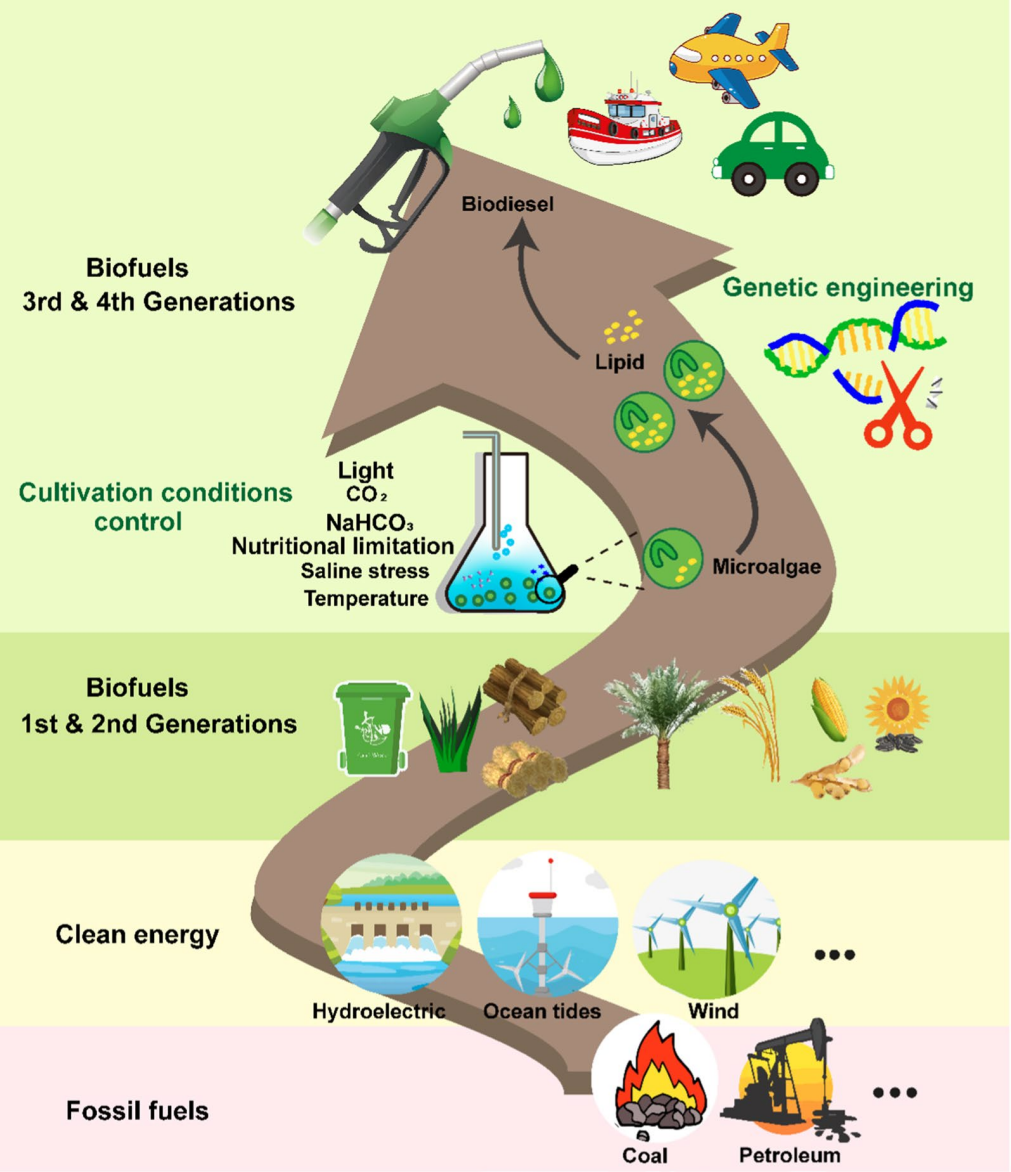

**Supplementary Information:**

The online version contains supplementary material available at 10.1186/s13068-024-02461-0.

## Highlights

- Highly adaptable microalgae strains are the preferred choice for large-scale biofuel production.
- Omics technologies and genetic editing have significantly increased lipid accumulation in microalgae, but rigorous risk assessment is required.
- Adjusting the parameters of PBRs can lead to efficient lipid accumulation.
- A promising future for the microalgae biofuel industry is highly anticipated in China.

**Supplementary Information:**

The online version contains supplementary material available at 10.1186/s13068-024-02461-0.

## Background

For several decades, increasing global population, rapid industrialization, and urbanization have led to a significant rise in energy demand. In 2021, primary energy use surged by 5.8%, predominantly from fossil fuel [1]. The availability of secure, affordable, and low-carbon energy is of increasing importance, given the declining reserves of fossil fuels, the global warming crisis, and the geopolitical tensions that exist around the world [2]. Currently, various forms of clean energy, such as solar, ocean tides, hydroelectric, geothermal, wind, and biofuels, are being researched and implemented by numerous countries to satisfy the increasing energy demands [3, 4]. Among them, biodiesel stands out as the only biofuel capable of replacing fossil fuels, while other clean energies are constrained by their ecological footprint, economic performance, dependence on the environment, and geographical location, making them more suitable for power generation and heating purposes only [5]. Hence, biofuel production is receiving global attention and holds promising prospects for development and future demand. In terms of global biofuel production in 2021, besides the traditional biofuel producers such as the United States, Brazil, and the European Union, India, Switzerland, and Argentina also made noteworthy contributions to the growth in biofuel production [1]. Simultaneously, there is a continuous emergence of new national-level policies encouraging the gradual substitution of fossil fuels with biofuels [6, 7]. As one of the developing countries, China faces the problem of fossil fuels shortage and carbon emission. In recent years, China is also striving to find a sustainable development path of energy, and the biofuel industry is considered to be an important means to ensure energy security. The present situation and policy support of biofuel industry in China are introduced in this paper.

**Fig. 1:**
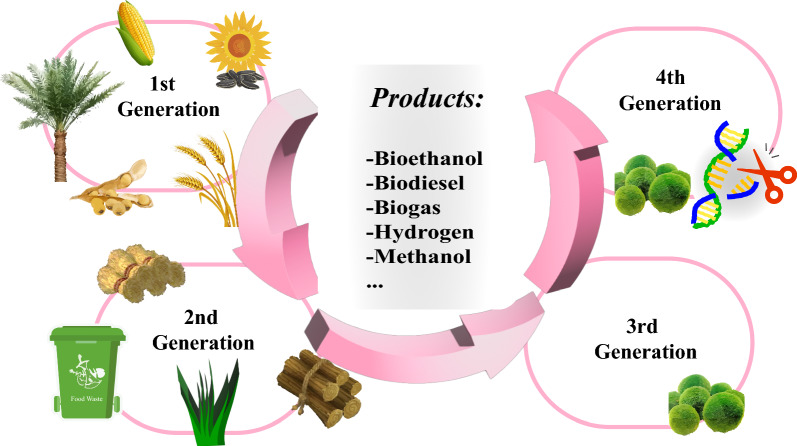
Overview of feedstocks and productions of different biofuel generations. 1st generation biofuels utilize corn, soybeans, wheat, sunflower oil, and palm oil as feedstocks; 2nd generation biofuels utilize switchgrass, poplar, straw, and food waste as feedstocks; 3rd and 4th generation biofuels utilize microalgae as feedstocks, with microalgae being editable in 4th generation

Biofuels, derived from a diverse range of sources, such as higher plants, algae, microorganisms, crop straw, and livestock manure, offer a wide array of product types, including biodiesel, biohydrogen, bioethanol, biogas, and solid fuels. They are typically divided into four generations based on the feedstock sources (Fig. 1). The first-generation of biofuels often utilizes edible materials, such as corn, soybeans, wheat, sunflower oil, and palm oil as feedstocks, which can result in competition between food and fuel sources [8]. Second-generation biofuels, primarily derived from non-edible sources, such as switchgrass, poplar, straw, and food waste, are more sustainable than the first generation. However, both face limitations due to grain, land, and water resources [9].

Microalgae, which are important feedstocks for third- and fourth-generation biofuels, can grow in seawater, wastewater, food waste, and even saline–alkaline soil, and can obtain higher biomass than land plants using limited nutrients [10–15]. Microalgae exhibit a fast growth rate, a higher photosynthetic efficiency, and a better land utilization rate, which can yield a range of biofuel products [3, 16]. In the past two decades, scientists have made significant strides in researching microalgae biodiesel. Several species, including *Chlorella vulgaris*, *Nannochloropsis oceanica*, *Dunaliella salina*, *Botryococcus*, *Desmodesmus*, *Neochloris*, *Scenedesmus*, and *Tetraselmis*, have been identified as suitable for biodiesel production, with some of them producing biodiesel in laboratory scale with properties meeting ASTM standards [17–24]. *Synechococcus* strains also exhibit compliance with the EN 14214 standards in terms of iodine value and cetane number while also demonstrating lower cold filter plug point values [25]. Moreover, the fuel properties of diesel and microalgae biodiesel mixture have been extensively studied [26–28]. While various studies have documented methods of producing lipid from microalgae by manipulating culture conditions, fundamental limitations cannot be overcome if unsuitable strains are chosen for biofuel production. It is essential to conduct thorough investigations into species-specific characteristics regarding lipid production from microalgae. Hence, the selection of robust strains that could harvest a high amount of desired products is a crucial step in the large-scale production of biofuels [29], and this article will provide a comprehensive review on this topic.

Due to differences in cell size, lipid content and fatty acids (FAs) composition among microalgae species, not all of them are useful for biofuel production. Recently, genetically engineered microalgae have attracted the interest of researchers, and numerous species have been modified, including *Chlamydomonas*, *C. vulgaris*, *Phaeodactylum tricornutum*, *Thalassiosira pseudonana*, *Galdieria sulphuraria*, *B. braunii*, *D. salina*, etc. [30]. This approach employs genetic engineering to enhance and adjust the standardized traits of microalgae, resulting in the production of high-yield lipids. It proves to be more effective, precise, and rapid compared to random mutagenesis, offering extensive potential applications [31]. The resources, methods, and limitations of microalgae genetic engineering are all discussed in this article.

While the market potential for microalgae biofuels has been widely recognized, several economic, biotechnological, and environmental challenges must be addressed before microalgal products can enter the global market [32]. These include cost-effective dehydration and harvesting methods, process improvements in product extraction, as well as the thorough exploration of genomic resources and genetic tools [33, 34]. Thus, the objective of this paper is to outline the development direction of microalgae biofuels, taking into account the current achievements and challenges.

## Selection and cultivation of microalgae strains

### Types of microalgae from biofuel sources

The success of large-scale microalgae culture relies heavily on the selection of appropriate species. It should be noted that the lipid productivity of microalgae, which is a critical criterion for strain selection, has been a focus in previous studies. The type of lipids synthesized by microalgae can affect the properties of biodiesel [35]. Triacylglycerol (TAG) is the preferred lipid component for use as biodiesel feedstocks, and is usually expressed as the total lipid content as a percentage of dry weight [36]. While a recent study challenges the notion that natural cyanobacteria lack neutral lipid TAG, it remains a fact that TAG content in cyanobacteria is generally low [37, 38]. Unlike the abundant diacylglycerol (DG) found in cyanobacteria, TAG constitutes the primary lipid component in green algae [39, 40]. Moreover, some strains of cyanobacteria have been reported to possess higher lipid productivity, although it generally remains lower than that of green algae [41–43]. Therefore, the focus of this study primarily lies in green algae.

Green algae (Chlorophyceae) have the largest group of oleaginous microalgae, with high lipid abundance, and are good potential biodiesel feedstocks [37, 44, 45]. Typically, the average total lipid content of oleaginous green algae is 25.5%, while nutrient deficiency or stress conditions can increase the total lipid content substantially (up to 45.7%) [37]. The lipid content in many oleaginous green algae significantly surpasses that of typical lipid-producing crops, such as maize, soybean, canola, castor, sunflower, and palm oil. This high lipid content contributes to significant biodiesel production. However, for most microalgae, high biomass does not necessarily equate to high lipid productivity, and high lipid content may actually lead to lower biomass productivity. Consequently, it is not accurate to concentrate solely on lipid content and not biomass, since lipid productivity is determined by the combination of both factors. In the past decade, researchers have been striving to screen new microalgal strains to achieve higher lipid accumulation. Table 1 documents the biomass, biomass productivity, lipid content and lipid productivity of various green algae under different culture conditions. For *Neochloris oleoabundans* and *Botryococcus braunii*, the maximum lipid content can reach more than 74%, while their respective lipid productivity has not been the most impressive (Table 1). On the other hand, *Chromochloris zofingiensi*s displays the highest lipid productivity, as well as a high biomass productivity and lipid content, and seems to be a particularly suitable option for biodiesel production (Table 1). Lipid productivity reflects the actual situation of microalgal growth and lipid accumulation per unit time, avoiding the misleading conclusions that may arise from relying solely on lipid content or biomass productivity. The study by Yu et al. [46] also found that, under similar lipid content in *Chlorella*, accumulating more biomass per unit time can effectively reduce production costs. Therefore, comparing lipid productivity is a crucial step in strain selection for microalgal biofuel production.

**Table 1:** Biomass and lipid in various green algae species

Species	Biomass (g L^−1^)	Biomass productivity (mg L^−1^ d^−1^)	Lipid content (%, W/W_DW_)	Lipid productivity (mg L^−1^ d^−1^)	References
*Ankistrodesmus braunii*	1.43–1.57	ND	22.1–34.4	ND	[47]
*Ankistrodesmus falcatus*	1.4–2.29	340	16.49–53	56.07	[20, 47, 48]
*Ankistrodesmus fusiformis*	0.93–3.04	62.3–240	20.66–38.28	18.94–116.88	[20, 49]
*Botryococcus braunii*	0.5–2.74	104–250	20.4–74.4	48.3–112.43	[20, 50, 51]
*Chlamydomonas* sp.	2.49–6.09	240–280	4–46.2	36.17–380	[20, 52, 53]
*Chromochloris zofingiensis* (*Chlorella zofingiensis*)	8.3	171.4–691.67	30–64.5	36–445.7	[54–56]
*Chlorella protothecoides*	ND	ND	11.1–42.3	15.25	[57, 58]
*Chlorella sorokiniana*	ND	43–100	44	22.2	[59, 60]
*C. vulgaris*	2.83–3.47	234–730	11.9–54.2	30.2–204.91	[20, 57, 61, 62]
*Chlorococcum* sp.	ND	ND	20.77–38.9	22	[63, 64]
*Desmodesmus brasiliensis*	ND	130	17.99–24.2	23.39	[20, 65]
*D. salina*	1.23	50–150	18.9–25.1	10.26–36.48	[66, 67]
*Dunaliella* sp.	ND	150	22–33	33–39.3	[67]
*Dunaliella tertiolecta*	1.30	81	18–50	8.4–20.2	[68, 69]
*Haematococcus pluvialis*	0.57–0.79	80.71–112.14	13–35.9	ND	[70, 71]
*Nannochloris* sp.	0.17–0.75	18.1–83.44	26.29–53.13	29.21–59.03	[13]
*Neochloris oleoabundans (Ettlia oleoabundans)*	ND	386.5–413.1	13.1–74.5	46.7–172.6	[72–76]
*Raphidocelis subcapitata (Pseudokirchneriella subcapitata)*	ND	80	16.3–28.43	22.74	[20, 77]
*Scenedesmus dimorphus*	0.75–1.16	44–68	15.14–29.15	15.449	[78]
*Scenedesmus incrassatulus*	0.99–3.95	ND	19.7–37.7	ND	[47]
*Tetradesmus obliquus (Scenedesmus obliquus)*	0.5–1.5	50–160	16.73–55	26.77	[20, 79–81]

In addition to lipid productivity, the FAs composition is also essential for the success of biodiesel production as they can affect the characteristics of biodiesel. Microalgae FAs consist of both saturated and unsaturated FAs, featuring hydrocarbon chains ranging from 4 to 36 carbons [82]. The main saturated fatty acids (SFA), monounsaturated fatty acids (MUFA), and polyunsaturated fatty acids (PUFA) in microalgae are palmitic acid (C16:0), oleic acid (C18:1), linoleic acid (C18:2) and linolenic acid (C18:3), respectively [83]. Table S1 is not a strict comparison of the performance among 16 oleaginous microalgal species with FAs compositional profiles reported in the literature, and provides the percentage of each FAs to the total. Comparison with the FAs profiles of different microalgal species reveals several interesting features. First, SFA and MUFA are dominant in most of the algae examined. The sum of SFA and MUFA of *A. fusiformis*, *B. braunii*, *Chlamydomonas* sp., *C. vulgaris*, *Nannochloris* sp., *N. oleoabundans*, *S. incrassatulus*, *T. obliquus,* and *P. subcapitata* were more than two-thirds of the total FAs (Additional file 1: Table S1). Among these, the content of MUFA is considered an optimal choice for microalgal biodiesel production [84]. Second, all of these oleaginous microalgae have considerable amounts of C16 and C18 species. The C16–C18 of 16 microalgae accounted for 89.24% of the total FAs on average (Additional file 1: Table S1). Moreover, these oleaginous microalgae commonly exhibit elevated levels of C16:0 and C18:1, which are the most prevalent components in biodiesel [85]. Therefore, the selection of suitable strains for biodiesel production should take into account the FAs composition in microalgae, contributing to enhanced biodiesel performance.

### Factors affecting lipid accumulation

**Fig. 2:**
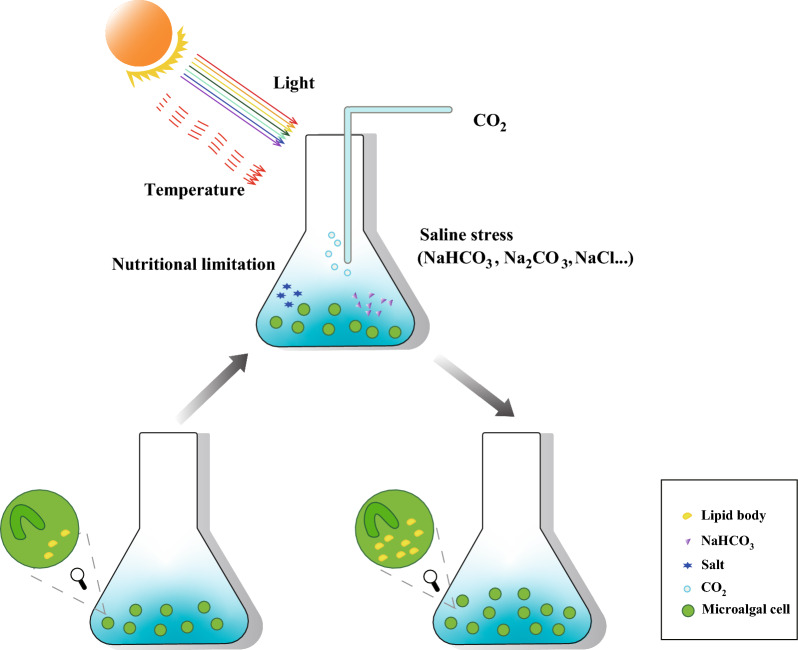
Methodology of enhancing lipid accumulation in microalgae cells. Nutrient deficiency, saline stress, light, temperature, CO_2_, and NaHCO_3_ can induce lipid accumulation in microalgae

Although oleaginous microalgae are abundant in nature, basal lipid levels can vary substantially by modifying lipid metabolism in different ways. Common approaches to increased lipid productivity include nutrient deficiencies, extreme stress, and environmental perturbations. This paper summarized the effects of inorganic carbon sources (CO_2_ and NaHCO_3_), nitrogen and phosphorus limitation, saline stress, light, and temperature on the lipid accumulation of microalgae (Fig. 2).

#### CO_2_ and NaHCO_3_ concentration

Phototrophic microalgae can produce biomass by absorbing CO_2_. Published data showed that 1–5% CO_2_ concentration is appropriate for numerous microalgae growth; however, higher CO_2_ concentration will inhibit the growth of microalgae [86, 87]. In this concentration range, increased CO_2_ concentration helps *Tribonema minus* [88], *Chlamydomonas* [89], and *Nannocholoropsis oculate* [90] to accumulate more lipids and achieve excellent lipid production. At the same time, excessively low or high CO_2_ concentrations lead to a reduction in lipid content. This implies that the optimal levels of CO_2_ affect lipid production and their subsequent accumulation within the cellular structure. In large-scale production, industrial flue gas is an economical source of CO_2_ for microalgae biofuel production, with CO_2_ concentrations typically ranging from 10% to 20% [91]. Currently, certain microalgae species have been discovered thriving in elevated CO_2_ environments, with a few demonstrating enhanced lipid accumulation capabilities even under a 15% CO_2_ culture condition [91–95]. Currently, Chile and Colombia have already applied microalgae photobioreactors for carbon capture [96], providing feasibility for the large-scale application of high-CO_2_-adaptive microalgae in utilizing flue gas for biofuel production. Although, it is important to note that these microalgae are merely a limited selection of representatives.

NaHCO_3_ is a valuable carbon source for some microalgae and is much easier to handle than gaseous CO_2_ in storage, transportation, and processing. In recent studies, exogenous NaHCO_3_ has been found to significantly increase the total lipid content of *D. salina*, *D. tertiolecta*, *Scenedesmus,* and *Nannochloris*, and the addition of NaHCO_3_ under nutrient deficiency conditions has further significantly increased the lipid content of *D. salina* [13, 97–99]. Besides, some microalgae even prefer NaHCO_3_ to CO_2_ for lipid biosynthesis [100]. This may be attributed to the enhanced activity of key enzymes in lipid metabolism due to the presence of NaHCO_3_, stimulating microalgae to assimilate extracellular inorganic carbon. In our previous study, we observed that the addition of exogenous high concentrations of NaHCO_3_ led to a significant accumulation of lipid bodies in microalgae, and the gene UTP-glucose-1-phosphate uridylyltransferase 3, associated with lipid synthesis, was upregulated [101]. Similarly, high concentrations of NaHCO_3_ increased the activity of Ribulose-1, 5-bisphosphate carboxylase/oxygenase (Rubisco), promoting the generation of 3-phosphoglycerate, which serves as a substrate for FAs production [102]. Another interpretation suggests that NaHCO_3_ enhances the photosynthetic capacity of microalgae, causing more carbon to be directed towards lipid accumulation. Indeed, in *Nannochloris* sp., we observed a significant enhancement in photosynthetic capacity after NaHCO_3_ treatment [13]. Furthermore, Roya et al*.* has also suggested that NaHCO_3_ increases lipid content while concomitantly enhancing the rate of CO_2_ fixation in *Chlorella* sp. [100]. In the selection and breeding of microalgae, the carbon fixation efficiency of high concentration inorganic carbon sources can be used as one of the screening criteria in the future.

#### Nutritional limitation

Numerous researchers have used nutritional limitation as an applied and promising strategy to alter and control the cell cycle and biochemical pathways associated with lipid production and accumulation in microalgae [103]. Nitrogen and phosphorus are regarded as fundamental factors that exert a direct influence on lipid accumulation in microalgae [104]. In particular, nitrogen deficiency is often cited as a booster of lipid augmentation in most microalgae species [105]. Nitrogen deficiency serves as a significant stimulus for lipid accumulation in various microalgae species, such as *Acutodesmus dimorphus* [106], *Nephrochlamys yushanlensis* [107, 108], *C. vulgaris* [109], *Scenedesmus quadricauda* [110], and *N. oceanica* [111]. These reports imply that the degradation of nitrogen compounds is the possible reason for lipid accumulation under nitrogen deficiency conditions.

During the initial stage of nitrogen deficiency, the photosynthetic capacity of microalgae decreases, and the excess carbon is more easily converted into storage compounds, such as lipids and starch. According to this view, the chlorophyll and proteins are degraded as a nitrogen source to support photosynthesis with nitrogen limitation, and the excess carbon is used to the synthesize FAs, which is also the reason for the changes in FAs composition of microalgae under nitrogen deficiency [94]. It is known that the FAs compositions of *N. oceanica* [112], *Coelastrella multistriata* [113], *Scenedesmus* sp. and *C. sorokiniana* [114], *Nannochloropsis gaditana* and *Coccomyxa elongata* [115, 116] indeed altered under nitrogen deficiency. In general, these changes involved notable increases in SFA and MUFA, accompanied by a decrease in PUFA. A decrease in PUFA and a simultaneous increase in the SFA resulted in a reduced degree of unsaturation of the total FAs pool. This may be related to oxidative damage caused by nitrogen deficiency, to which PUFA is particularly sensitive [117]. It is also possible that the production of enzymes involved in desaturation and elongation reactions is reduced at nitrogen-limited condition, leading to a decrease in PUFA production [118].

However, the growth of microalgae cells under nitrogen deficiency is inhibited usually, which is manifested as a decrease in chlorophyll content and yellowing cultures [119]. The intracellular nitrogen quota plays a crucial role in regulating the allocation of carbon between the functional pool and the storage pool, thus nitrogen deficiency consistently impacts the carbon flow in microalgae, as indicated in references [120, 121]. In the initial stages of the nitrogen-depleted condition, both carbohydrates and lipids are produced at higher levels. Nevertheless, as nitrogen levels decrease, the fixed carbon undergoes a shift from the synthesis of functional biomass to the generation of storage molecules, resulting in the redirection of previously stored carbon towards neutral lipids [121]. In that case, neutral lipid levels are significantly higher than carbohydrates [122]. Despite the unfavorable conditions that nutrient deficiency creates within microalgae cells, the prospect of increased lipid production and accumulation as a desirable outcome has motivated researchers and the industry to continue utilizing this technique [123].

Numerous investigations have explored phosphorus deficiency techniques in different strains to comprehend and optimize different output parameters. It has been found that phosphorus deficiency can increase the accumulation of lipids and FAs [113, 119, 124], and the changes of FAs composition were similar to that under nitrogen deficiency. However, the increase of lipids and FAs in phosphorus deficiency is much smaller than that in nitrogen deficiency [108, 125]. These results indicate that nitrogen deficiency is more effective than phosphate deficiency in inducing lipid accumulation in some microalgae species.

#### Saline stress

Saline stress has proven to be a highly effective method for enhancing lipid content, making it crucial to investigate lipid accumulation in microalgae as model organisms under saline stress in the context of biofuel development strategies. The lipid content of various microalgae species such as *C. reinhardtii*, *A. dimorphus*, *C. vulgaris*, and *Acutodesmus obliquus* has been found to increase when exposed to concentrations exceeding 0.1 M NaCl [66, 126–128]. It has been observed that microalgae respond to saline stress by reallocating resources to carbohydrates, which are later utilized in the biosynthesis of neutral lipids [129]. It should be emphasized that in the lipid accumulation process of microalgae, KCl, MgCl_2_, and CaCl_2_ also play crucial roles, and in certain species, they may exert a more pronounced influence than NaCl [130]. Moreover, saline stress not only increases the lipid content but also causes changes in the FAs profile [131, 132]. Although some microalgae showed an increase in MUFA, most microalgae still showed an increase in SFA after high concentration of NaCl treatments [128, 129, 133]. A previous study has shown that the FAs composition is closely related to the fluidity of cell membranes [134]. A decrease in unsaturated FAs is thought to help reduce membrane fluidity, which is critical for cells to withstand saline stress [135]. In conclusion, saline stress to a certain extent can promote microalgae to accumulate lipids. However, this promotion depends on the salt tolerance of microalgae.

#### Light and temperature

Light intensity is an essential parameter that plays a crucial role in the lipid content and FAs composition of microalgae. A lack of sufficient light can lead to reduced production, while excessive light can result in photoinhibition. Microalgae exposed to high light intensity (HL) lead to excellent lipid and TAG accumulation [136–141]. It has also been observed that HL conditions can induce an increase in lipid content in *Synechocystis* sp. PCC6803 [142]. Although excessive light causes oxidative damage to cells, cells respond to the situation by converting the light energy into lipids for storage within the cell. Hence, strains with high light-tolerance are ideal for large-scale production systems in outdoor conditions. Study of two high-light-tolerant *Chlamydomonas* mutants supported this claim, showing that their lipid productivity increased twice as much as that of the wild type under high-light conditions [143].

From the early fluorescent lamps to the light emitting diodes (LEDs) used today, artificial light sources for microalgae culture vary in the characteristics of the emission spectrum. The light spectrum is not only essential for microalgae growth but also critically affects lipid accumulation [144]. Several studies have specifically investigated the impact of LED light on the enhancement of lipids in microalgae, highlighting the significant role of blue light in this process [137, 145, 146]. Nevertheless, it is worth noting that certain microalgae have demonstrated sensitivity to green light, resulting in either a substantial inhibition or promotion of lipid accumulation [147, 148]. Moreover, the FAs composition of microalgae is also affected by the different light spectra [149–151]. These results highlight the potential of coupling light spectrum to lipid synthesis in bioprocess development.

Temperature is another primary environmental driver affecting growth and lipid content in microalgae cells; however, different species have different ranges of adaptation to temperature. The mechanisms that govern and regulate the adaptation of physiological responses to temperature stress are largely unknown [152]. For example, in *Tetraselmis subcordiformis*, *N. oculata* and *Parachlorella kessleri*, the response temperature of lipid accumulation of each microalga is different [153, 154]. Besides, a decrease in the culture temperature leads to the shift of desaturases activity and an increase in the unsaturation level in FAs, which ensure the cell membrane fluidity under temperature stress conditions [155–158]. These findings suggest that the lipid content and FAs composition of microalgae can be influenced to a certain extent by temperature control. However, it is important to note that the method of inducing lipids through temperature control is species-specific and challenging to implement on a large scale in production.

In summary, achieving high productivity for biofuels hinges on the rational control of factors, such as light, temperature, stress, and nutrition. When cultivating microalgae outdoors, prioritizing those with high light tolerance is crucial, and considering their salinity and alkali tolerance is also essential. This ensures that microalgae can thrive in extreme environments without interference from foreign microorganisms, a fundamental requirement for large-scale biofuel production using microalgae. However, for indoor cultivation, microalgae with a high tolerance for CO_2_ are preferred, as they can strike a balance between carbon sequestration and lipid production. As mentioned earlier, such microalgae can utilize industrial flue gas for biofuel production, further reducing costs and enhancing sustainability.

### The cultivation systems of microalgae to induce lipid accumulation

**Fig. 3:**
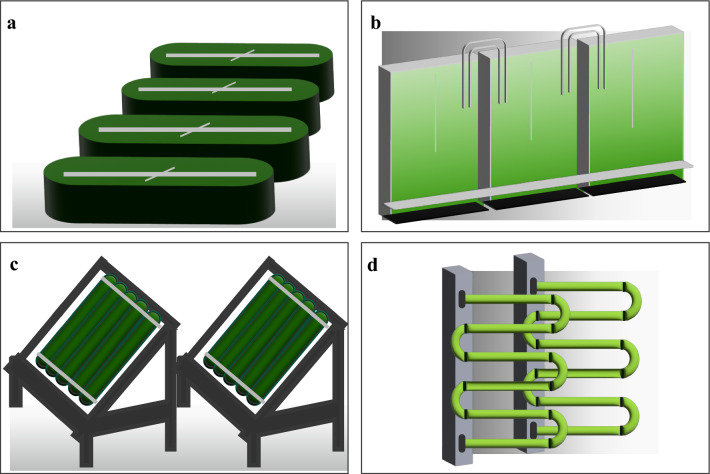
Microalgae cultivation systems. **a** Open cultivation systems, **b** flat plate photobioreactor, **c** column photobioreactor, **d** tubular photobioreactor

Cultivation of microalgae appears to be relatively straightforward and can be established with limited or even no supervision, using simple nutrients and even waste-water while absorbing CO_2_ from the atmosphere [159]. Large-scale microalgae cultivation can occur in either open or closed systems (Fig. 3). Open systems encompass a range of setups, including small artificial ponds and large bodies of open water, each employing various agitation systems [160]. Closed system mainly refers to various types of PBRs, including plastic bags, horizontal or vertical tubes, bubble columns, airlift reactors, flat panel tubular reactors, plate reactors, and even membrane-based systems [83]. Initially, open systems were more acceptable than PBRs from a commercial point of view, as they were relatively economical to build, could accommodate large areas and were easy to operate [5]. However, such systems are unable to control the stable environmental conditions (temperature and light), and are inherently susceptible to contamination, leading to inefficient utilization of light by the cells, contamination by predators, loss due to evaporation, and escape of CO_2_ [161]. Due to the shortcomings of open systems, much attention has been paid to closed PBRs. On one hand, PBRs prevent evaporation and reduce both contamination risks and CO_2_ losses [162]. On the other hand, PBRs have strong photosynthetic efficiency leading to a high biomass and lipid productivity [163].

#### Performance of PBRs

PBRs allow for a wider selection of microalgae species, while open systems typically lead to the cultivation of only a few robust photoautotrophic species [160]. Microalgae such as *C. reinhardtii*, *N. oceanica*, *Chlorella pyrenoidosa*, *Golenkinia* sp., and others have been successfully cultivated in PBRs. Some of them exhibit superior growth performance in PBRs compared to open systems, showcasing the ability to achieve higher biomass and lipid accumulation levels [164–167]. Since the design of different PBRs directly affects biomass productivity, novel PBRs to improve the process design and operation are rising. Studies focused on how to optimize biomass production, improve contaminants removal efficiency, reduce cost and space dominance by renovating the configurations of PBRs [163, 168]. Among them, studies focused on enhancing lipid production and quality through membrane PBRs predominantly aim to utilize wastewater for inducing lipid production [169–172]. This approach is considered more environmentally friendly in terms of water resource management compared to other types of PBRs.

Unlike closed systems, PBRs can improve the efficiency of light energy utilization by microalgal cells through their unique light path design. The overall optical conditions in PBRs can be evaluated in terms of light distribution and light penetration. As an example, the utilization of light and mass transfer efficiency of microalgae in flat-plate PBRs can be significantly enhanced by optimizing the ratio of the gap in the baffle opening to the width of the bioreactor [173]. By doing so, microalgae can efficiently convert optical energy into chemical energy, leading to the efficient accumulation of biomass and lipids. In fact, studies have reported that microalgae cultivated in these improved PBRs have achieved nearly threefold increases in biomass and lipid accumulation [174]. Besides, hydrodynamics is another crucial factor that affects the performance of PBRs. Proper fluid mixing helps prevent sedimentation, influences nutrient delivery, and governs the movement of microalgal cells in the reactor, which in turn impacts their light exposure history [175]. In one study, four different PBRs were investigated at varying air flow rates to evaluate their impact on biomass and lipid production from *N. oleoabundans*, and it was found that the bubble column reactor with a ring sparger displayed superior performance [176]. By adding mixers and improving sparger performances, novel PBRs with efficient mixing, high rates of gas–liquid mass transfer, and well-defined fluid flow patterns can be obtained for microalgal lipid accumulation and biofuel production purposes [177–180]. The results suggest that the production of microalgal biofuels in PBRs is influenced by the reactor configuration and operating conditions. Ideally, PBRs should not cause cell disruption and damage, but cell damage occurs in PBRs (with turbulent flow) when the smallest eddies are the size of cells or much larger than the cells. Thus, optimizing the performance of PBRs is an essential technical approach to improve microalgae biomass and lipid production.

#### Limitations and future perspectives of PBRs

While many alternative configurations have been shown to be effective for microalgae growth, the major drawback is the high-power consumption and other operating costs of PBRs. In an effort to compare each type of PBRs, Vo et al*.* [181] summarized their disadvantages as follows. For example, flat plate PBRs require more space and land, have photoinhibition effects and dark zone formation, leading to high construction and energy costs. For column PBRs, which prefer internal illumination and are more costly to mix, the surface-to-volume ratio is generally lower. The utilization of tubular PBRs poses the challenge of preventing system overheating and fouling, as well as high operational costs. Similarly, soft-frame PBRs are prone to issues such as fragile materials, expensive components, culture leakage, and poor mixing resulting from the formation of dead zones. Despite being regarded as the most promising cultivation system in recent years, hybrid PBRs systems also face problems, such as membrane fouling and negative energy balance.

If the performance of the outdoor lipid production process is described in terms of productivity and yield, and the calculation is based on ground area, then theoretically the parameters obtained from the pilot plant can be applied to the full-scale plant. However, microalgae batch lipid production pilot tests are usually conducted in single plate reactors, which means that the application of these parameters to full-scale plants is difficult [182]. Naturally, without consistent data on both biomass productivity and lipid productivity, it becomes challenging to determine which types of PBRs are better suited for microalgae biofuel production. The optimal designs of PBRs are contingent upon the microalgae species being used and the intended final metabolite. There are multiple parameters within PBRs that still require optimization to improve lipid production. It is clear that sustainable development and scaling of PBRs will be the central focus of future research.

## Genetic engineering of microalgae to increase lipid production

Microalgal energy density is relatively low. This is due to the fact that microalgal cells are mainly composed of water, proteins, lipids, sugars, etc., with water accounting for a large proportion, and proteins and lipids being relatively scarce in comparison. This leads to a need for greater input to produce the same amount of energy, which in turn reduces the unit value of the product, indirectly increasing the production cost. It is worth mentioning that genetic engineering can cut the cost of microalgal biofuels by 15–20% [183]. Most studies using genetic engineering to induce lipid accumulation have concentrated on either mutagenizing a single target gene or engineering multiple genes. In general, genes are either knocked out or knocked in to achieve desired outcomes, and they are often of great importance in lipid metabolism. Modifying metabolic pathways involves the up-regulation or down-regulation of transcriptional and translational genes [184]. An example of genetic engineering in microalgae is the modification of *N. oleoabundans* by introducing an endogenous key enzyme called diacylglycerol acyltransferase 2 (NeoDGAT2). It was observed that overexpression of *NeoDGAT2* led to an increase in TAG content and productivity in *N. oleoabundans* [73]. Target-specific knockout of the phospholipase A2 gene in *C. reinhardtii* improved overall lipid production by up to 64.25% [185]. Overexpressing Malonyl CoA-acylcarrier protein transacylase gene and glycerol-3-phosphate acyltransferases gene promoted neutral lipid accumulation in microalgae, too [186, 187]. Unlike green algae, cyanobacteria are prone to stress incurred due to the synthesis of biofuel compounds [188]. Therefore, the focus of the genetic engineering of cyanobacteria is the transfer of biofuel compounds. In *Synechocystis* sp. PCC6803, overexpression of the thioesterase gene has been shown to stimulate the significant secretion of free FAs into the extracellular environment [189, 190]. Similar findings have also been observed in *Synechococcus elongatus* PCC7942 and *Synechococcus* sp. PCC7002 [191, 192]. Researchers have been working tirelessly to develop new genetic engineering strategies to further enhance lipid accumulation in microalgae. Table 2 shows some key features of the genetic engineering of microalgae for enhanced biofuel production. It can be observed that genetic editing is driving microalgae towards a positive direction in biofuel production. However, this requires a thorough understanding of microalgae metabolic pathways. The development of omics provides abundant background information for targeted production of microalgae products, while the evolution of genetic engineering tools also offers technical support in reducing the cost of microalgae biofuel production.

**Table 2:** Biotechnological modifications of microalgae for enhanced biofuel production

Species	Type of modification	Targeted gene	Result	Reference
*C. reinhardtii*	Nuclear transformation; Heterologous expression; CRISPRi-based transcriptional silencing; CRISPR–Cas9-based target-specific knockout; Overexpression; Gene knockout	Acetyl-CoA synthetase (ACS2); Acyl-ACP thioesterase (DtTE); Phosphoenolpyruvate carboxylase (PEPC1); Phospholipase A2 (PLA2); Phosphorus stress response 1 (PSR1); DNA-binding-with-one-finger (Dof, crDOF)	Neutral lipid, total lipid, FA content and lipid productivity increased	[185, 193–199]
*C. sorokiniana*	Overexpression and transcriptomics	Carbonic anhydrase (CA); rbcL and accD	Lipid productivity increased	[200, 201]
*Mortierella alpina*	Construction and overexpression CCMP1545 (MpFADS6)	Delta-6 desaturase (FADS6)	Eicosapentaenoic acid (EPA) content increased	[202]
*N. oceanica*	Overexpression and knockdown	Malonyl CoA-acylcarrier protein transacylase (MCAT); Type-1 diacylglycerol acyltransferase (NoDGAT1A); Type-2 diacylglycerol acyltransferases (DGAT2); Fatty acid desaturases (FAD); bZIP1 transcription factor (NobZIP1)	Neutral lipid, SFA, MUFA, PUFA and EPA content increased	[186, 203–208]
*N. salina*	Overexpression of transcription factor; Heterologous expression	Basic helix–loop–helix (NsbHLH2); Basic leucine zipper transcription factors (NsbZIP1); AP2 type TF Wrinkled1 (NsAtWRI1)	Total lipid and FA content increased	[209–211]
*P. tricornutum*	Overexpression	Glucose-6-phosphate dehydrogenase (G6PD); Malic enzyme (PtME); Glycerol-3-phosphate acyltransferase (GPAT1); Lysophosphatidic acid acyltransferase (LPAT1)	Total lipid and neutral lipid content and productivity increased	[212–214]
*S. obliquus*	Overexpression	Type-2 diacylglycerol acyltransferse (DGTT1)	Total lipid content and productivity increased	[215]
*Schizochytrium* sp.	Overexpression	Malonyl-CoA: ACP transacylase (MAT); Malic enzyme; Codon-optimized ELO3 gene	Total lipid, PUFA and Docosahexaenoic acid content increased	[216, 217]

### Types of genetic engineering resources in microalgae

#### Algomics resources

Omics techniques for algae, known as ‘algomics’, including transcriptomics, genomics, proteomics, metabolomics, and metagenomics, prove to be highly valuable in assisting the design of experiments and predicting potential interactions and outcomes in algae research [218]. The integration of omics methodologies expands the scope of research for addressing stress-related challenges in lipid production. The combination of these omics approaches and bioinformatics tools and databases will be crucial in realizing economically viable microalgal-based biofuels. Furthermore, the integration of omics resources enables microalgae to produce a wider range of metabolites and bioactive compounds, accelerating the discovery and utilization of new microalgal species resources.

Genome sequencing and analysis play a vital role in comprehending and understanding microalgal systems [103]. The complete genome sequence of the cyanobacterium *Synechocystis* sp. PCC6803 was published as early as 1996, providing precise genetic information for gene modification [219]. In contrast, research on the genomes of eukaryotic microalgae started later. In the beginning, the whole genome sequence of microalgal genome projects have been generated only for a limited species, namely, *C. reinhardtii*, *T. pseudonana*, and *P. tricornutum*, but the number of publicly available microalgal sequenced genomes is escalating currently [220, 221]. Investigators have used insights from the genome of *C. reinhardtii* to perform functional annotation of lipid biosynthetic genes in microalgae [222]. By genome editing, the gene UDP-glucose pyrophosphorylase was disrupted and a 45-fold of TAG accumulation was found in *P. tricornutum* [223]. With the development of next-generation sequencing technology, genome mining of microalgal strains has become much more economical and reliable. Recently, an initiated genome sequencing project called 10KP aims to sequence the genomes of at least 1000 green algae (microalgae and macroalgae) as well as 3000 photosynthetic and non-photosynthetic protists [224]. As the number of published microalgae genome sequences increases, the evolution and adaptation of microalgae can be unraveled, and metabolic engineering strategies can be employed to reduce wet lab work. Besides, there are three web-based resources available for algal genomics, including pico-PLAZA (http://bioinformatics.psb.ugent.be/pico-plaza/), AlgaePath (http://algaepath.itps.ncku.edu.tw), and ALCOdb (http://alcodb.jp). These genomics resources provide genomic information, intuitive tools for functional genomics, gene co-expression data, and details of gene expression prediction based on metabolic pathways in some microalgae [225–227]. To this end, genome editing tools have been added to develop or increase the productive efficiency of biofuels and other valuable products in sustainable and renewable ways. Insertion, deletion, mutation, gene knockdown, and gene knockout are common strategies used in genome editing [228].

Transcriptomics is a valuable tool that can reveal details about gene expression by analyzing RNA transcripts, providing insights into the functions of various microalgae strains. Transcriptomic data are particularly beneficial as it offers a comprehensive reference data set for the discovery of new genes and the construction of metabolic models through computational analysis. The Marine Microbial Eukaryote Transcriptome Sequencing Project has conducted transcriptome sequencing on almost 700 marine microbial species, including 140 microalgae species [229]. Subsequent to sequencing, decontamination, pooling, and dereplication methods have been employed to refine these sequences, rendering them more amenable to analysis, with lower levels of contamination, redundancy, and improved balance in the resulting phylogenies [230]. In recent years, analysis of gene expression involved in lipid production at the transcriptome level in microalgae has offered valuable insights into metabolic pathways, as well as contextual information regarding modifications at the genomic level in algae [231–234].

In contrast to transcriptomics, proteomics is concerned with regulatory pathways at the post-transcriptional level. Proteomics provides quantitative data on protein expression under varying experimental conditions, helping to elucidate how protein expression can be upregulated or downregulated in response to environmental conditions that can affect both the production of a specific product and the survival of the cell [235]. Proteomic studies have identified a variety of proteins involved in lipid enhancement. In the case of *N. oculate*, it has been observed that organic carbon and nitrogen derived from the breakdown of proteins and pigments are primarily directed towards the FAs synthesis [236]. Nonoyama et al*.* [237] reported the detection of membrane trafficking-related proteins from the proteome involved in TAG hydrolysis in oleaginous diatoms. A proteomic analysis of *C. zofingiensis* has shown that structural proteins are present in high abundance and that enzymes involved in lipid metabolism work in concert with other lipid metabolism-related enzymes localized in ER [54]. The proteins involved in lipid biosynthesis and in FAs oxidation have been up-regulated under hypersaline conditions in *Microchloropsis gaditana* [238]. Furthermore, 29 putative lipid droplet-localizing proteins of *Aurantiochytrium limacinum* have been identified despite proteomics and lipidomics analyses, and approximately half of the proteins were accounted for by thraustochytrid-specific lipid droplet protein 1, which was thought as a structural protein in the lipid droplet [239]. There were certainly studies that combine the results of transcriptomic and proteomic analyses to analyze the molecular mechanisms of lipid accumulation in microalgae, and the availability of quantitative data under defined experimental conditions provided strategic insights for strain improvement in microalgae [240, 241].

Metabolomics focuses on the analysis of low molecular weight compounds involved in the maintenance of cellular biological processes, to target processes or pathways and explain their mechanisms. Similar to transcriptomic and proteomic studies in microalgae, it is also desirable to investigate lipid metabolism under different environmental conditions though metabolomic to achieve efficient lipid production. Up-regulated metabolic fluxes associated with increased lipid biosynthesis were found in a comparative time course metabolomics analysis of a *C. sorokiniana* mutant [242]. Metabolomics was used to identify the biochemical changes induced by antibiotics in *S. obliquus*, and it was found that norfloxacin triggers the shift of additional common precursors from the starch pathway to the FAs synthesis pathway, resulting in a shift in carbon allocation from carbohydrates to lipids [243]. Moreover, the metabolomic analysis has indicated that the intermediates in glycolysis and the tricarboxylic acid cycle contribute to the accumulation of astaxanthin and lipids in *Haematococcus pluvialis* treated with melatonin [244]. Metabolomics has been proven to be instrumental in microalgal research, but there is no dedicated database for microalgal metabolomics [33].

The advancing research in algomics is opening up new possibilities for microalgae biofuel production. Particularly, the integration of multi-omics approaches allows for a comprehensive understanding of the metabolic networks in microalgae. This approach reveals intricate regulatory relationships among key genes, proteins, and metabolic products, enabling precise control of production pathways and the attainment of desired end-products, such as lipids.

#### Mutant resources

Forward genetics can generate extensive pools of mutant phenotypes without prior knowledge about the genetics and metabolism of the target organism through random mutagenesis and adaptive laboratory evolution [245]. The use of random mutagenesis to obtain microalgal strains with the desired phenotype is widespread. Through methods, such as ^60^Co γ-ray, UV exposure, and chemical mutagenesis, mutants of *N. gaditana* [246, 247], *N. oculate* [248, 249] and *Scenedesmus* sp. [250, 251] have been successfully generated. These mutants have exhibited robust abilities for lipid accumulation. However, random mutagenesis is non-specific, unstable and even lethal or at least detrimental to the mutagenized organisms [245]. Therefore, appropriate selection methods, such as fluorescence-activated cell sorting, are needed to prevent phenotypic reversion. Similar to random mutagenesis, adaptive laboratory evolution is another cost-effective approach used for improving microalgal strains. In this model, microalgae cells are subjected to continuous selective pressure for a long period to obtain desired traits. As mentioned earlier in this study, microalgae can induce high lipid production under nutrient deficiencies, saline stress, high light and temperatures, and examples of how microalgae adapt to these stresses will not be mentioned here.

Insertional mutagenesis necessitates the presence of a DNA transformation protocol specific to each organism, as well as the identification of the mutation site associated with a particular mutant phenotype by the presence of selective markers and/or genetic or phenotypic tags [252]. Metabolism can be diverted to the lipid synthesis pathway by insertion and integration into the microalgal genome of a foreign DNA sequence present with a selective marker [252]. Mutant libraries are good tools for studying the function of uncharacterized genes and are also very important for reverse genetic research. Insertional mutagenesis by random non-homologous end joining is the preferred method of choice for generating mutant libraries [253]. The studies found an increase in FAME and lipids in the insertional mutants of both *N. salina* and *N. oceanica* [254, 255]. However, due to the lack of efficient transformation and genetic manipulation protocols, the application range of microalgae mutant libraries are far less wide than that of *Arabidopsis thaliana* and yeast [33]. At present, there are 2 random insertion mutant libraries of *Chlamydomonas*. Chlamydomonas Resource Center (www.chlamycollection.org/products/clip-strains) applied over 62,000 insertional mutants of *C. reinhardtii* that covers 83% of nuclear protein-encoding genes. Institute of Hydrobiology (Chinese Academy of Sciences) built an indexed mutant library, including ~ 150,000 *C. reinhardtii* insertional mutants [256]. As an important microalgae resource, the establishment of mutant libraries still needs to solve the problems of efficient identification of insertion sites.

### Methods of genetic engineering in microalgae

#### Transformation techniques

Genetic engineering encompasses several steps, including transformation, which involves the introduction of foreign DNA into the target cell. This process entails the penetration of DNA into the cell, the integration of the foreign DNA into the genome of the target cell, and finally, the stable and desired expression of the integrated DNA [257]. The efficacy of transformation is highly contingent upon the microalgal species, and it is necessary to carefully choose both the genetic modification and transformation methods based on the specific species and type of modification [103]. Several transformation methods have been utilized to introduce DNA into microalgal cells, including the use of artificial transposons, electroporation, particle bombardment, sonication, viruses, bacterial infection, silicon carbide whiskers, glass bead-based agitation, and agrobacterium-mediated transformations [258]. Among them, the glass bead-based transformation was performed early in *C. reinhardtii* [259], and the discovery of cell wall-deficient mutants made transformation easier to achieve. Electroporation and particle bombardment are considered to have the highest transformation efficiency, and neither method requires prior removal of microalgal cell walls [260]. Currently, some examples have been observed to increase the content of neutral lipids and total lipids in microalgae by electroporation and particle bombardment [207, 261, 262]. Furthermore, agrobacterium-mediated transformation results in a higher rate of false-positive transformants, thus it does not have a significant advantage over electroporation methods in microalgae. Other methods of DNA delivery, such as agitation with silicon carbide whiskers, artificial transposons or sonication, have been reported, but they have not been widely adopted for lipid accumulation.

#### Genome editing tools

Novel genome-editing tools, such as RNA interference (RNAi), zinc-finger nucleases (ZFNs), transcription activator-like effector nucleases (TALENs), and clustered regularly interspaced short palindromic repeats (CRISPR/Cas9), have been employed to improve the quality and quantity of desired products in microalgae [184]. RNAi is a powerful tool used for gene silencing and modulating gene functions [184]. Levitan et al*.* [263] used RNAi to knock down the expression of the nitrate reductase gene in *P. tricornutum* to obtain a larger lipid content. In the diatom *T. pseudonana*, strains with a targeted knockdown of a multifunctional lipase/phospholipase/acyltransferase have been shown to exhibit growth patterns similar to the wild type and demonstrate increased lipid content under both continuous light and alternating light/dark conditions [264]. ZFNs is an initial tool for genome editing in microalgae and has been used successfully in *C. reinhardtii* in recent years, but is rarely used to study lipid accumulation [265–267]. Although TALENs is feasible for enhancing lipid production in microalgae [268], similar to RNAi and ZFNs, it is still limited by their tedious design steps and high off-target events, so CRISPR–Cas9 came into being [269, 270].

In the CRISPR–Cas9 system, the Cas9 nuclease creates double-stranded breaks in DNA sequences that are determined by the guide RNA, making microalgal genome editing more efficient, accurate and simple [271, 272]. A significant amount of effort has been invested in mitigating off-target mutations and cytotoxicity related to the use of Cas9 ribonucleoproteins. Researchers have established CRISPR–Cas9-based targeted gene knockout methods in microalgae, such as *C. reinhardtii* and *N. oceanica* [267, 273]. In addition, this method has been used to explore ways to enhance lipid accumulation in microalgae and has been demonstrated in *N. gaditana* [274], *Tetraselmis* sp. [275], *P. tricornutum* [276], and other microalgae. The rapid pace of technological progress provides hope that CRISPR/Cas will soon be established as a routine and trustworthy technique for genome editing in any type of microalga.

### Limitation and risk of genetic engineering microalgae

As previously noted, there have been numerous experiments and studies conducted on the genetic engineering methods applied to various microalgal species. The application of genetic engineering in microalgae holds immense potential for boosting lipid production. An issue with working with GM microalgae is the potential environmental and ethical impacts of their release. Unlike other GM organisms that are cultivated in controlled environments for high-value products, GM microalgae in outdoor cultivation systems can escape into the environment through means, such as wind, water, birds, animals, natural disasters, or accidents [277]. This could have unintended consequences that are difficult to predict and control. Therefore, several factors should be taken into consideration when evaluating the potential negative ecological effects of GM microalgae and their impact on the environment and human health. These factors include potential changes to food web structure, displacement of native species such as phytoplankton, local extinctions, harmful algal blooms, and serious societal, cultural, and economic impacts in the case of toxic strains [278]. To mitigate the potential risks associated with the use of GM microalgae, both regulations for their use and strict biosecurity measures are necessary to ensure the safe importation of both foreign GM and wild-type species into the local environment. Besides, there is a need for the development of ‘suicide genes’ to control the spread of hazardous algae strains in case of an accidental release into the environment.

Another significant challenge is the compatibility of transgenic microalgae with large-scale cultivation, as the conditions required for their growth and cultivation may be difficult to replicate at a larger scale. Upon exposure to the conditions of large-scale cultivation, the strains encounter a wider range of conditions that differ from the controlled laboratory environment, which in turn affects their transgenic properties. Tabatabaei et al*.* [279] highlighted that lower growth rates and poor gene quality are significant challenges in genetic engineering that could hinder the commercialization of algae on a global scale. In addition, some of the genetic engineering techniques used for microalgae, such as homologous recombination, can result in long and complex constructions, which may impact the stability and functionality of the modified microalgal strains. As a result, their productivity in outdoor cultivation is often not as high as it is under optimized laboratory conditions. To date, no reports of outdoor cultivation of GM microalgae have been made due to the various risks and uncertainties associated with open cultivation [280].

## Microalgal biofuel production opportunities in China

China is the largest energy-consuming country in the world. In 2021, China consumed 157.65 exajoules (EJ) of primary energy, an increase of 10 EJ from 2020 [1]. The rapid economic growth has led to an increasing demand for energy in China, thus the need to enhance the development and utilization of renewable energy and strive towards energy transformation. According to the report, the consumption of renewable energy in China increased by 32.9% in 2021 compared to 2020, indicating efforts made by China in energy transformation [1]. It is expected that the share of renewable energy in China will reach 13% by 2030 [281]. Biofuel is one of the important types of renewable energy, which has been paid attention to in recent years. The main types of biofuels in China include biodiesel, bioethanol, biogas, and biohydrogen, among others. In 2021, the consumption of biofuels was 48 thousand barrels of oil equivalent per day, with a biofuels production of 64 thousand barrels of oil equivalent per day [1]. Despite the increase in the production and consumption of biofuels in China, the country still has a long way to go in comparison with the United States, Brazil, and the European Union, which have set more ambitious targets and made greater progress in incorporating biofuels into their energy mix. In addition, the Chinese government is implementing measures to advance the cultivation and utilization of biofuels and has instituted a number of policies. In 2014, the National Energy Administration of China issued the ‘Biodiesel Industry Development Policy’, which proposed exploring and optimizing microalgae cultivation and lipid extraction processes, to achieve a technological breakthrough in microalgae biodiesel production. In 2016, the ‘CHINA: 13th Five-Year Plan for Biomass Energy Development’ proposed promoting the large-scale development of biogas and steadily developing biomass power generation. In 2017, the Chinese government issued a policy to expand the production of bioethanol and promote the use of ethanol gasoline for vehicles. The ‘2021 Work Plan for Biomass Power Generation Project Construction’ stated that the total central subsidy for biomass power generation in 2021 is CNY 2.5 billion. China aspires to harness the potential of biofuels and other sustainable energy sources to fulfill its expanding energy needs, strengthen its energy security, decrease its reliance on non-renewable fuels, and decrease its carbon emissions. Presently, China’s utilization of biofuels on a commercial scale remains largely at the second-generation level, with third- and fourth-generation biofuels mainly in the research stage [262, 282, 283]. Table 3 lists several large-scale bioethanol production companies in China and their production capacity. These companies mainly use materials, such as grains to produce bioethanol. However, like the problems faced by global biofuels, China also needs to adjust its reliance on grains for biofuels to alleviate the pressure on the food supply. Therefore, large-scale production of third- and fourth-generation biofuels using microalgae as feedstock is the key challenge that China needs to overcome in the future.

**Table 3:** List of bioethanol producing industries in China

Company	Location	Capacity (10 Kilo-tons per Year)	Feedstock
Henan Tianguan Enterprise Group Co., Ltd	Nanyang, Henan	70	Corn and wheat
JILIN FUEL ALCOHOL COMPANY LIMITED	Jilin, Jilin	60	Corn and wheat
Anhui BBCA Biochemical Co., Ltd	Bengbu, Anhui	60	Corn and wheat
COFCO Bio-Energy (Zhaodong) Co., Ltd	Zhaodong, Heilongjiang	40	Corn and wheat

It is undeniable that Chinese scientists have made great progress in the field of microalgal biofuel research, including the development of microalgal strains with high lipid content, exploration of cost-effective large-scale production methods, and improvement of microalgal lipid extraction processes, among others [19, 68, 88, 140, 173, 206, 208, 276]. In the future, with the continuous improvement of technology and the reduction of costs, the microalgal biofuel industry in China is expected to experience rapid development. At the same time, government policy support and the entry of industries are also important factors. Therefore, the prospects of microalgal biofuels in China are very optimistic.

## Future outlook

Substantial progress has been made in the selection and cultivation optimization of lipid-producing microalgal strains in the past decade. However, significant improvements must be made to the economic viability of current microalgal biofuel production to compete favorably with fossil fuels before ultimately replacing them [284]. Enhancing microalgae dehydration and harvesting techniques can help achieve maximum biomass yield at minimum cost. The continual enrichment of genome sequences and mutant libraries, along with the improvement of gene editing tools and synthetic biology, will provide strong support for the development of microalgal potential as a renewable energy source. The development of novel techniques for lipid extraction and purification is a key area that requires significant progress in downstream processing. For example, the feasibility discussion on the application of AI models in the fucoxanthin extraction and quantification process provides valuable insights [285]. In addition, exploring the simultaneous production of biofuels and other high-value byproducts, as well as the use of microalgal biofuels in aerospace and other fields, are also promising directions for the future. Overall, the future of microalgal biofuels hinges on the developing cost-effective methods for the most efficient technology, which will accelerate the commercialization of this type of biofuel.

Undoubtedly, microalgae constitute a vast family with a diverse classification. This discussion has only scratched the surface, focusing primarily on the research and applications of eukaryotic microalgae in biofuel production. In addition, this article primarily emphasizes strategies for enhancing lipid accumulation in microalgae and does not delve into the processes of harvesting, extraction, purification, and other production processes. As a result, there are certain limitations to consider.

## Conclusions

Microalgae biofuel presents an effective solution to the fossil fuel crisis, but it faces challenges in cost-effective large-scale production. The first step towards large-scale production is selecting robust and adaptable microalgae strains. While controlling cultivation conditions and adjusting PBRs parameters can influence the yield and performance of microalgae biofuel products, gene editing and multi-omics technologies offer more precise production routes. However, it is crucial to consider the environmental and human health risks associated with large-scale outdoor cultivation of GM microalgae. China still has significant work ahead to achieve large-scale production of microalgae biofuels, but the convergence of technology and market demand promises a bright outlook. In the future, the application of synthetic biology holds the potential to reduce the risks associated with GM microalgae and enhance the economic viability of their biofuel production.

### Supplementary Information

**Additional file 1: Table S1**. Fatty acids composition of green algae (percentage of total fatty acids).

## Data Availability

The data sets used and/or analysed during the current study are available from the corresponding author on reasonable request.

## Abbreviations

DG: Diacylglycerol
TAG: Triacylglyceride
FAs: Fatty acids
SFA: Saturated fatty acids
MUFA: Monounsaturated fatty acids
PUFA: Polyunsaturated fatty acids
C16:0: Palmitic acid
C18:1: Oleic acid
C18:2: Linoleic acid
C18:3: Linolenic acid
Rubisco: Ribulose-1, 5-bisphosphate carboxylase/oxygenase
HL: High light intensity
LEDs: Light emitting diodes
PBRs: Photobioreactors
NeoDGAT2: Diacylglycerol acyltransferase 2
RNAi: RNA interference
ZFNs: Zinc-finger nucleases
TALENs: Transcription activator-like effector nucleases
CRISPR/Cas9: Clustered regularly interspaced short palindromic repeats
GM: Genetically modified
EJ: Exajoules
